# Robust manipulation of magnetism in La*A*O_3_/BaTiO_3_ (*A* = Fe, Mn and Cr) superstructures by ferroelectric polarization

**DOI:** 10.1107/S205225251801624X

**Published:** 2019-01-15

**Authors:** Dong Chen, Guangbiao Zhang, Zhenxiang Cheng, Shuai Dong, Yuanxu Wang

**Affiliations:** a Institute for Computational Materials Science, School of Physics and Electronics, Henan University, Kaifeng 475000, People’s Republic of China; b Institute for Superconducting and Electronic Materials, Australian Institute of Innovative Materials, University of Wollongong, Wollongong NSW 2500, Australia; c School of Physics, Southeast University, Nanjing 211189, People’s Republic of China

**Keywords:** multiferroic layers, interfaces, magnetism, metal–insulator transitions, ferroelectric polarisation, perovskite oxide superstructures

## Abstract

Robust control of magnetism is both fundamentally and practically meaningful and highly desirable, although it remains a big challenge. In this work, perovskite oxide superstructures LaFeO_3_/BaTiO_3_ (LFO/BTO), LaMnO_3_/BaTiO_3_ (LMO/BTO) and LaCrO_3_/BaTiO_3_ (LCO/BTO) (001) are designed to facilitate tuning of magnetism by the electric field from ferroelectric polarization, and are systemically investigated via first-principles calculations. The results show that the magnetic ordering, conductivity and exchange interactions can be controlled simultaneously or individually by the reorientation of the ferroelectric polarization of BTO in these designed superstructures. Self-consistent calculations within the generalized gradient approximation plus on-site Coulomb correction did not produce distinct rotations of oxygen octahedra, but there were obvious changes in bond length between oxygen and the cations. These changes cause tilting of the oxygen octahedra and lead to spin, orbital and bond reconstruction at the interface, which is the structural basis responsible for the manipulation. With the G-type antiferromagnetic (G-AFM) ordering unchanged for both ±*P* cases, a metal–insulator transition can be observed in the LFO/BTO superstructure, which is controlled by the LFO thin film. The LMO/BTO system has A-type antiferromagnetic (A-AFM) ordering with metallic behavior in the +*P* case, while it shifts to a half-metallic ferromagnetic ordering when the direction of the polarization is switched. LCO/BTO exhibits C-type antiferromagnetic (C-AFM) and G-AFM orders in the +*P* and −*P* cases, respectively. The three purpose-designed superstructures with robust intrinsic magnetoelectric coupling are a particularly interesting model system that can provide guidance for the development of this field for future applications.

## Abstract 2

Robust control of magnetism in La*A*O_3_/BaTiO_3_ (*A* = Fe, Mn, Cr) perovskite superstructures is realized. Not only magnetism switching but also a spin-polarized 2D electron gas is created by switching of the polarization. A powerful scheme to realize robust control of both magnetism and the 2D electron gas is demonstrated. This work highlights the direction of further development in the multiferroic field.

## 1. Introduction   

Traditional information storage media, *e.g.* hard disks, which consist of non-magnetic and magnetic layers formed on a support, feature slow reading/writing speeds, high energy consumption and weak thermal shock resistance. Utilization of the magnetoelectronic coupling in multiferroic materials might provide an alternative approach to solving these problems by electrically writing magnetic bits with extremely low energy consumption. Unfortunately, ferromagnetism and ferroelectricity are naturally contradictory in their requirements for 3*d* transition metals, and it is difficult for them to coexist in single-phase materials. So far it has not been possible to identify a stable, single-phase multiferroic material in which the magnetization can be totally switched. The artificial superstructure, with its combination of materials that are abruptly different in their properties, provides an ideal platform to directly couple the different physical properties between adjacent layers or create new physical properties, such as magnetoelectric coupling, superconductivity, multiferroicity, colossal magneto-resistance, *etc*. (Dong, Yu *et al.*, 2009 ▸; Zhai *et al.*, 2014 ▸; Dong, Yamauchi *et al.*, 2009 ▸). In recent years, there has been extensive research focused on perovskite oxide superstructure materials in the hope that one part of the interface can provide magnetism and the other can provide ferroelectricity (Weng *et al.*, 2016 ▸) as a result of chemical compatibility as well as similar lattice constants of the constituent perovskite oxides (Weng *et al.*, 2015 ▸). In particular, some perovskite superstructures are composed of two compounds with respective ferroelectric polarization and ferromagnetism, which provide an ideal scheme for the possible electric field control of magnetism with reduced energy consumption; they are also suitable systems for the study of interface effects (Bousquet *et al.*, 2008 ▸).

Actually, the robust control of magnetism by an external electric field has already been realized in non-perovskite metals. Fechner *et al.* (2012 ▸) have demonstrated a 180° switching of the magnetization in a PbTiO_3_/Fe/Au/Fe heterostructure, which can be mainly attributed to magnetoelectric coupling at the PbTiO_3_/Fe interface. This coupling is amplified by interlayer-exchange coupling in the Fe/Au/Fe trilayer. There are also many ways to control the magnetism for perovskite materials such as LaMnO_3_ (LMO). The magnetization of an LMO thin film grown on SrTiO_3_ (STO) (Kim & Christen, 2010 ▸; Roqueta *et al.*, 2015 ▸) or LaAlO_3_ (Zhang *et al.*, 2017 ▸) substrates can be directly controlled by changing the oxygen partial pressure. Two phase transitions occur in the LMO thin film (Hou *et al.*, 2014 ▸), namely the transition from the A-type antiferromagnetic (A-AFM) phase to the insulating ferromagnetic phase and then to the metallic ferromagnetic phase. Gibert *et al.* (2015 ▸) investigated the LMO/LaNiO_3_ heterostructures and found that the interface-driven magnetic moment variations have a strong dependence on interface reconstructions. A robust ferromagnetic moment and large room-temperature magnetoresistance are demonstrated by the LMO thin films (Vila-Fungueiriño *et al.*, 2015 ▸). To date, a variety of methods to control magnetism in layered perovskite heterostructures and superstructures have been considered. Wang *et al.* (2015 ▸) synthesized high-quality ultrathin LMO films on TiO_2_ terminated STO (001) substrates. An atomically sharp transition from the non-magnetic phase to the ferromagnetic phase can be observed when the thickness of LMO reaches five unit cells, which is argued to be the result of charge reconstruction induced by polar discontinuity. This ferromagnetic ordering is generated by the self-doping effect (*i.e.* electrons are transferred from the surface to the interface), which contradicts previous theoretical results (Hou *et al.*, 2014 ▸; Dong *et al.*, 2008 ▸; Lee *et al.*, 2013 ▸). Through an optical second-harmonic generation experiment, Mishina *et al.* (2016 ▸) found that the magnetic configuration remained the same for the situation with or without an external electric field applied on a La_0.7_Ca_0.25_MnO_3_/BaTiO_3_ superstructure. Some theoretical and experimental results show that a change in the interface magnetism can be achieved by switching the ferroelectricity. For example, the control of magnetism and conductivity via an external electric field has been demonstrated theoretically in La_1−*x*_ *D* *_x_*MnO_3_/BaTiO_3_ (001) (*D* = Ca, Sr and Ba) interfaces (Burton & Tsymbal, 2009 ▸). Unfortunately, the control of magnetism via an external electric field is rarely observed in metallic materials since the electric field cannot penetrate more than a few unit cells before it is completely screened by conductive layers. On the other hand, the technique of magnetism manipulation by ferroelectric polarization has developed quickly. Duan *et al.* (2006 ▸) investigated the control of magnetism of the Fe/BaTiO_3_ multilayers. Only the magnitude of the magnetic moment, and not its magnetic ordering, is changed when the direction of the ferroelectric polarization alters. Dong & Dagotto (2013 ▸) have also investigated the control of magnetism through the ferroelectric polarization of BaTiO_3_ (BTO). The origin of the magnetization control is the modulation of charge density induced in the interfacial layers to screen the polarization charges of BTO. In recent work, the polarization control of magnetization has been experimentally demonstrated in the La_2/3_Sr_1/3_MnO_3_/BaTiO_3_ superstructure (Cui *et al.*, 2015 ▸). This manipulation of magnetism is mainly due to the interfacial orbital reconstruction of the superstructure, driven by the shuttle displacement of Ti atoms under ferroelectric polarization. The conductance of different La_1−*x*_Sr*_x_*MnO_3_ heterostructures can be dramatically switched by the switching of ferroelectric polarization (Yin *et al.*, 2013 ▸). The highly spin-polarized MnO_2_ layers near the interface act as an atomic scale spin valve in series with the ferroelectric tunnel barrier, which creates a switch for the conductance. To date, only metallic materials (not insulating ones) have been considered for the control of magnetism by BTO. The experimental demonstration of tuning effects in the above superstructure provides a model system showing the effectiveness of tunneling effects imposed by ferroelectric polarization.

Since the conclusions of the above-mentioned experimental and theoretical investigations are different and still the subject of debate, it is highly desirable to analyze the modulation of magnetism and its underlying mechanism in perovskite superstructures. The modulation of magnetism in artificially designed thin films and superstructures is crucial to their implementation in magnetoelectronic devices (Takamura *et al.*, 2013 ▸). Therefore, novel physical phenomena can only be observed near the interface, and the electric field has only a limited tuning effect on the whole system. Designing a novel superstructure system and making a real sample for experimental examination is very costly and time consuming, and more importantly, it is not generally applicable in many cases. Fortunately, first-principles modeling and calculations allow us to precisely control the superstructure structure, polarization and magnetism on an atomic level, and simulate the tuning of magnetism by electric polarization before real samples are fabricated and examined (Huang & Dong, 2014 ▸). Our scheme is to directly control the magnetic moments of the magnetic atoms that are tuned by ferroelectric polarization, thus realizing the robust manipulation of the magnetism.

BaTiO_3_ is an important material because of its ability to maintain strong electric polarization that can be reoriented easily by an electric field (Callori *et al.*, 2012 ▸). We chose tetragonal BTO (space group *P4mm*) for several reasons: (i) it is non-toxic compared with the popular ferroelectric material PbTiO_3_; (ii) perovskites grown on BTO have attracted considerable interest since the BTO crystal can accommodate a large amount of lattice strain during epitaxial growth; (iii) BTO is a typical ferroelectric system with a strong spontaneous polarization of 27 µC cm^−2^ (Wei *et al.*, 2017 ▸), and its ferroelectric behavior can be easily and significantly tuned by, for example, Sr substitution for Ba to form a solid solution (Tabata *et al.*, 1994 ▸); and (iv) the strong ferroelectric polarization of BTO can be easily switched by application of an electric field, thereby realizing the robust manipulation of the magnetism of ferromagnets. The LaFeO_3_ (LFO, Néel temperature *T*_N_ = 740 K) (Acharya *et al.*, 2010 ▸), LaMnO_3_ (LMO, *T*_N_ = 140 K) (Murakami *et al.*, 1998 ▸) and LaCrO_3_ (LCO, *T*_N_ = 253 K) (Wang *et al.*, 2013 ▸) compounds are all antiferromagnetic insulators with interlayer antiparallel spin alignments. They all have the same orthorhombic structure (space group *Pbnm*) with a continuous crystal framework, but possess different properties. In particular, the high *T*_N_ indicates a strong superexchange coupling in the LFO bulk. The material compatibility of BTO and La*A*O_3_ (LAO, *A* = Fe, Mn, and Cr) makes the formation of the superstructures experimentally achievable, therefore we have selected perovskite superstructures formed from LAO and BTO layers as the focus of this study, and report robust full control of magnetism by polarization, demonstrating how the displacement of oxygen and octahedral tilting can affect the ferroelectricity, ferromagnetism and magneto-electricity in a class of LAO/BTO superstructures.

## 2. Results and discussion   

Our superstructures consist of single LAO (*A* = Fe, Mn and Cr) unit cells alternating with four BTO unit cells grown along the (001) direction (Dong & Dagotto, 2013 ▸). The lattice parameters *a*, *b* and *c* of the superstructures are fully optimized to obtain the ground state, and the results are given in Tables S2–S4 of the supporting information. On one hand, the ferroelectric titanate needs to be relatively thick to maintain its polarization; however, the ultrathin LAO components only involve bilayers, which polarization can effectively penetrate. We hope that robust control of magnetism can be realized experimentally when the bilayers are coupled to ferroelectric polarizations. It is worth noting that through modern digital synthesis techniques, such as laser molecular beam epitaxy, superstructures can be fabricated with layer thickness on the unit-cell level and with near-perfect interfaces on an atomic scale (*i.e.* with minimal roughness, no misfit dislocations or other defects observed), which opens up exciting opportunities for the design of novel materials with richer physics (Shah *et al.*, 2010 ▸). Thus, the design of our materials is experimentally practical.

In order to explore the robust control of magnetism when the La*A*O_3_ (*A* = Mn, Fe and Cr) bilayers are coupled to ferroelectric polarizations, density functional theory calculations were carried out to determine the electronic and magnetic properties of the different superstructures. More details of our Vienna *ab initio* simulation package *VASP5.3.5* (Kresse & Furthmüller, 1996 ▸) calculations can be found in the supporting information. Before the simulation of superstructures, it is essential to check the physical properties of bulk LaFeO_3_, LaMnO_3_ and LaCrO_3_, which is not a trivial task. The agreement between the calculated results and other works confirms the reliability of our calculation set up (Table S1).

**Figure 1:**
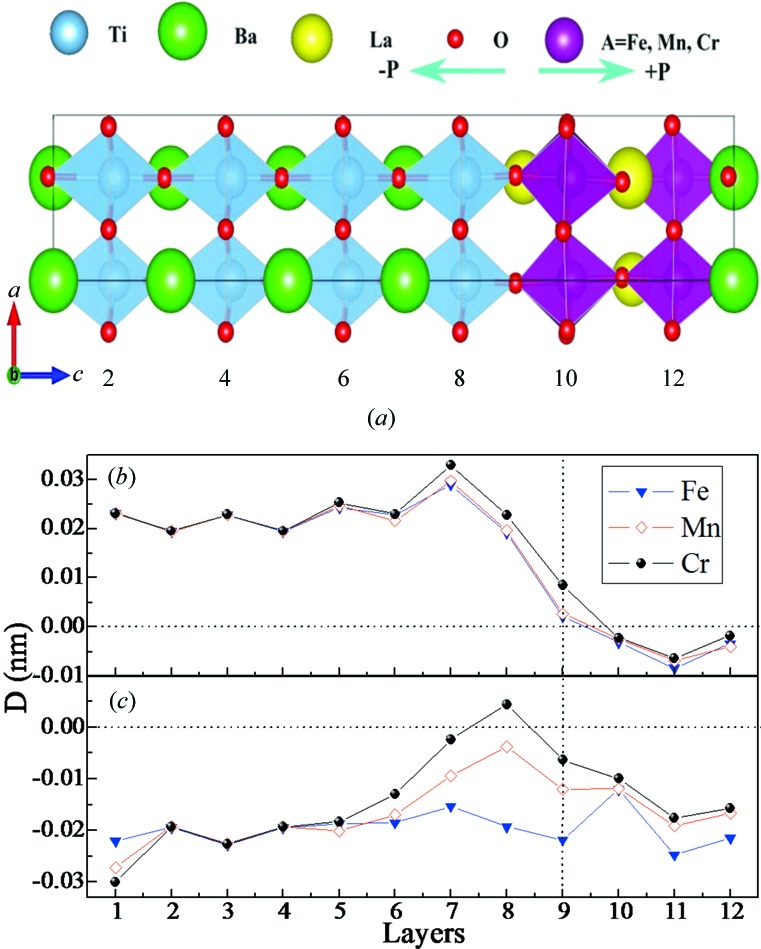
(*a*) Schematic structures of the LAO/BTO (*A* = Fe, Mn and Cr) superstructures. The arrows denote the directions of ferroelectric polarization. (*b*) The average local out-of-plane displacements between anions and cations for the −*P* case. (*c*) The average local out-of-plane displacements between anions and cations for the +*P* case.

By introducing four monolayers of LAO supercells (including two LaO and two *A*O_2_ layers) and eight monolayers of BTO supercells (including four BaO and four TiO_2_ layers), we built the LAO/BTO (*A* = Fe, Mn and Cr) superstructures, obeying the typical perovskite sequence within the 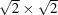 in-plane supercells along the (001) direction. The four monolayers of LAO and eight monolayers of BTO are shown in layers 1–8 and 9–12 of Fig. 1 ▸(*a*), respectively. Two asymmetric polar interfaces are hereby taken into account: the TiO_2_–LaO–AO_2_ and TiO_2_–BaO–AO_2_ layers are defined as the *n*- and *p*-type interfaces, respectively. When LAO is deposited on the BTO substrate, the ferroelectric polarization of BTO breaks the space-reversal symmetry, making the interfacial LAO layers partially polarized. In this work, two types of ferroelectric states with positive and negative polarizations have been adopted for full structural optimization and atomic relaxation. The polarization pointing from the *n*-type to the *p*-type interface is defined as the +*P* case, whereas −*P* corresponds to the case where the polarization points from LAO to BTO.

The layer-resolved local dipole *D*, which is defined as the average value of an oxygen atom (anion) displacement, relative to the metal atom (cation) perpendicular to the interface, is exhibited in Figs. 1 ▸(*b*) and 1(*c*). Oxygen octahedra rotations and tilts in varying degrees, together with the Jahn–Teller distortions can be found in the three superstructures, but are not the main reasons for the magnetism variations of the superstructures (see Table S5 and the following discussions in the supporting information). The polarization has enormous effects on the displacement of oxygens, namely, *D* is negative and positive for the +*P* and −*P* cases, respectively. As shown in Fig. 1 ▸(*b*), the three −*P* curves show wave-like characteristics in the BTO layers, and then decrease dramatically across the interface. Finally, the local dipoles became negative in the LAO layers. As shown in Fig. 1 ▸(*c*), the oxygens in BTO are displaced away from the interface (layer 9) under the +*P* condition, indicating a net polarization (in BTO) pointing to the interface. Interestingly, the +*P* curves increase slowly at first, and then demonstrate very different behavior near the interface: the LFO curve changes very slowly, while the LMO curve increases moderately and remains negative. The dipole of LCO goes through the positive region, but then drops back to the negative region. The average bond lengths between O and Fe, Mn and Cr atoms near the interfaces are 1.86, 1.84 and 1.85 Å, respectively. These values are slightly shorter than for bulk LFO (1.94 Å), LMO (1.95 Å) and LCO (1.96 Å) compounds, indicating stronger interactions between adjacent Fe/Mn/Cr and O atoms. The large oxygen shifts can be expected to determine the interface properties, because these atoms mediate the interaction between LAO and BTO. It is worth noting that the +*P* curves are almost twice as low as those of −*P* case in the LAO region. This agrees with the fact that +*P* will split the *A*O_2_ layer, thus enhancing the charge disproportionation. The mechanism leading to such variations is related to the interfacial chemical-bonding effect, which will be clarified below.

As shown in Table 1 ▸, the net magnetizations of the G-type antiferromagnetic (G-AFM) LFO/BTO and C-type antiferromagnetic (C-AFM) LCO/BTO are 0 and 0.03 μ_B_, respectively. Theoretically, these two net magnetizations should be zero. This difference is mainly due to the low symmetry that arises when we build the superstructures, which prevents the two Fe atoms in the same plane from being strictly symmetric. The difference in the Fe magnetic moment *m*_2_ for the opposing polarization directions reaches 0.07 μ_B_, which leads to a minute net magnetization change of *ΔM* = 0.01 μ_B_. The magnetic structure (G-AFM) of LFO/BTO is not affected by the ferroelectric polarization, which remains the same as that of bulk LFO. Correspondingly, LaFeO_3_ can be used as the substrate for magnetic storage devices. The net *M* of LaMnO­_3_ bilayers is switched from −0.01 to 3.82 μ_B_, accompanying the +*P* to −*P* switching. The ferromagnetic configuration displays a large *M*, giving rise to 99.7% modulation by switching the polarization. This is higher than the result (93.9%) for the La_0.75_Sr_0.25_MnO_3_/BTO system obtained by Dong & Dagotto (2013 ▸). Compared with Dong’s results, in our work, not only the net magnetization *M* itself, but also the magnetic ordering can be modulated. As shown in Table 1 ▸, the total energies indicate that the ground state of the LCO/BTO superstructure tends to form the G-AFM order under the −*P* condition. The most striking result is that the C-AFM state has the minimum energy under the +*P* condition. It can be seen that the reason why the G-AFM→C-AFM phase transition occurs is the increasing magnetic moments (*m*_2_) of the Cr atoms as the ferroelectric polarization reverses.

By using perovskite oxides with the same structure, we have observed the FM→AFM phase transition, the C-AFM magnetic order and an unchanged magnetic structure when the polarization direction changes in the LAO/BTO superstructures. Entirely different magnetic orders are obtained in these superstructures, which might be directly associated with hole accumulation (antiferromagnetic state) and depletion (ferromagnetic state) around the interfaces, thus corresponding to the strongest magnetoelectric effect. Robust control of the magnetic moments may be realized by the altered polarization of BTO, which can be easily realized by experimentation.

**Figure 2:**
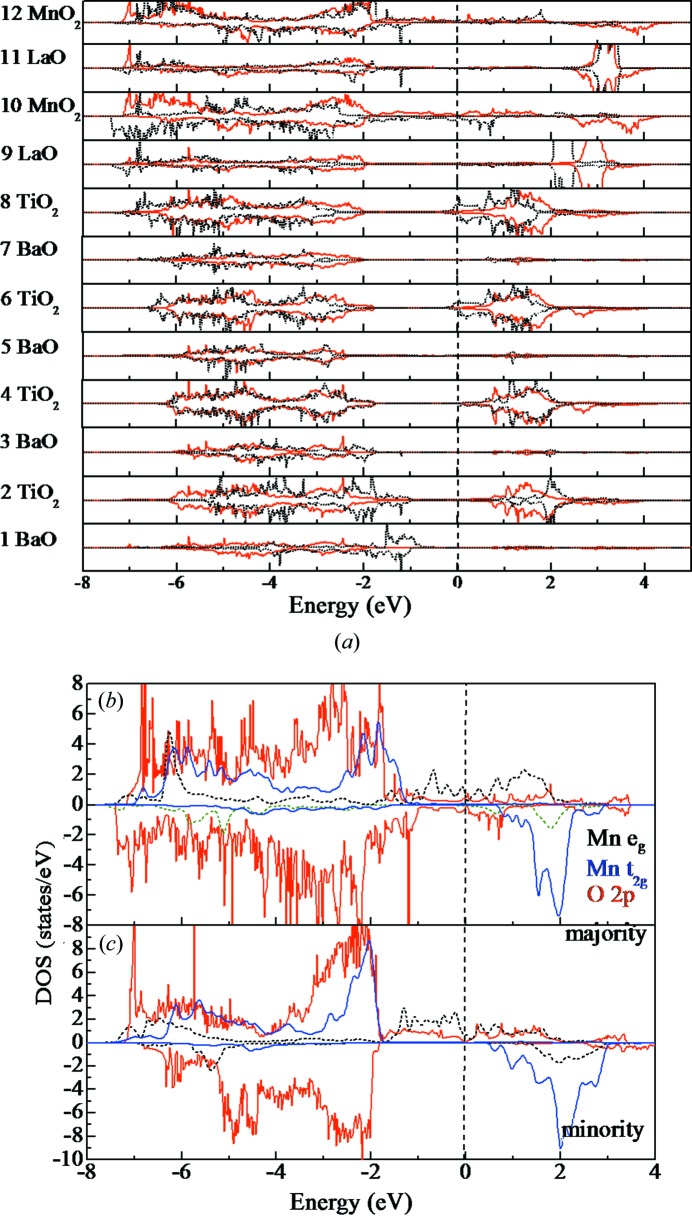
(*a*) The layer-resolved density of states of the LMO/BTO (001) superstructure (black line: +*P*, red line: −*P*). (*b*) The projected density of states of the LMO layers for the +*P* case. (*c*) The projected density of states of the LMO layers for the −*P* case. (The Fermi level *E*_F_ is located at 0 eV and indicated by the vertical dashed line.)

The layer-resolved density of states of the LMO/BTO (001) superstructure is presented in Fig. 2 ▸(*a*). For the *−P* case, the top of the valence band in both the LMO and BTO regions shows a downward shift to the lower-energy region when approaching the interface, indicating that the internal electrical field of the whole superstructure is greatly affected by the ferroelectric polarization. The electrons do not appear in the BTO region and are only found in the two MnO­_2_ layers, *i.e.* the electron generation is not ‘at’ but ‘beneath’ the TiO­_2_/LaO interface. When the polarization reverses from −*P* to +*P*, both the majority and minority states are shifted toward the higher energy region so that the hybridization is enhanced, since the Mn-3*d* and O-2*p* bands are more delocalized. In Fig. 2 ▸(*b*), the superstructure exhibits metallic conductivity, which is mainly due to the small but identifiable density of states at *E*_F_. The Mn atoms in the tenth and twelfth layers exhibit perfect half-metallic properties. It is clear that the valence band is mainly composed of Mn-3*d* states hybridized with O-2*p* states, while the conduction band near the *E*_F_ mainly consists of Mn-3*d* antibonding states with mixed O-2*p* antibonding states, as shown in the spin-down channels.

As shown in Figs. 2 ▸(*b*) and 2(*c*), the projected density of states reflects the coexistence of spin-polarized *e_g_* and *t_2g_* states at Mn sites, which are delocalized and broadly distributed through the valence band. With the partially occupied *e_g_* and *t_2g_* states, the contribution to the energy comes from both the superexchange and double-exchange interactions. In Fig. 2 ▸(*b*), strong interactions among the Mn-*e_g_*, Mn-*t_2g_* and O-2*p* states are observed in the spin-up channel. The superexchange interaction contributed by the *t_2g_* states may stabilize the antiferromagnetic phase of the LMO/BTO superstructure. In Fig. 2 ▸(*c*), there is a considerable number of *e_g_* electrons that appear around −5 eV. These states mainly originate from the chemical bonding between the Mn-*e_g_* and O-2*p* states. The *e_g_* density of states shifts toward the lower-energy region compared with that in Fig. S1(*b*), which means that the occupancy of *e_g_* electrons increases. The partially occupied *e_g_* states can mediate the double-exchange between the Mn-*t_2g_* core spins, which overcomes the antiferromagnetic superexchange, so that a ferromagnetic configuration is stabilized. As we can see in Fig. 2 ▸(*c*), a notable feature is that the minority-spin density of states is completely unoccupied at the Fermi level, which causes the half-metallic behavior. Only a flat majority-spin *e_g_* band exists near the *E*_F_. The bandwidth of the Mn-*t_2g_* state is narrower than for the Mn-*e_g_* state, which reflects the easier localization of *t_2g_* orbitals than the *e_g_* orbitals. Generally speaking, the half-metallic character is mainly contributed by Mn, while the BTO film makes almost no contribution to the half-metallic states. The LMO/BTO system is also semi-metallic, namely, the density of states at *E*_F_ tends to zero. This behavior is associated with a Dirac-cone-type band, which is demonstrated in our band structure analysis [Fig. S4(*c*)]. The layer-resolved and projected density of states are significantly changed from those of a non-spin polarized metal [Fig. 2 ▸(*b*)] to those of a half-metal [Fig. 2 ▸(*c*)]. In particular, the spin splitting density of states shown in the MnO_2_ layers indicates the ferromagnetic order of LMO/BTO in the *−P* case, which generates the magnetic moment.

As illustrated in Figs. 2 ▸(*b*) and 2(*c*), the destruction (+*P* case) of the half-metallic property is caused by the charge imbalance at the interface, in which the MnO_2_ layers lack electrons because the TiO_2_ layer does not donate electrons to the MnO_2_ layers. Therefore, the Fermi level *E*_F_ shifts to a higher energy, thus destroying the half-metallicity. The downward shift of the projected density of states corresponds to hole depletion, which is mainly due to the fact that the density of states at *E*_F_ decreases under the −*P* condition. The TiO_2_/LaO/MnO_2_ interface layers act as a magnetic switch to favor either the antiferromagnetic state (hole accumulation) or the ferromagnetic state (hole depletion) depending on the polarization orientation, which leads to a large variation in the magnetic moment and thus a large magnetoelectric effect. The whole LMO/BTO system is changed to a magnetic superstructure in the −*P* case because of the spin splitting of electrons. The LMO/BTO superstructure can be used as a sensing material for detecting harmful and toxic gases, since the O_2_ molecule is paramagnetic (Sobhan *et al.*, 2015 ▸). It can also be used as the photoanode for photocatalytic water splitting to recombine electrons and holes, as well as for separating photoelectrons from holes (Ji *et al.*, 2013 ▸).

The LFO/BTO superstructure remains G-AFM in both the ±*P* cases. No apparent 2D electron gas can be seen near the Fermi level in the +*P* case. A metal–insulator transition can be observed for the LFO/BTO system when the polarization of BTO reverses (see the density of states results in the supporting information, Fig. S1). For the LCO/BTO system, the metallic 2D electron gas can be observed near the *E*_F_ of the BaO/CrO_2_ interface in the +*P* case. For the *−P* case, the superstructure becomes a semiconductor. We found that the electronic and magnetic properties could be greatly affected by the different atomic environments and interface states. The whole LCO/BTO system is G-AFM (C-AFM) in the −*P* (+*P*) case, which agrees well with the results listed in Table 1 ▸ (see the density of states results in the supporting information, Fig. S2). In Figs. S3–S5, we show the band structures of different tetragonal superstructures with different performances. The LFO/BTO system is a metallic semiconductor with an indirect band gap of 1.12 eV in the *+P* and *−P* cases. The LMO/BTO system shows a topological feature.

Table 2 ▸ shows the number of electrons for the 3*d* orbitals (both *e_g_* and *t_2g_*) of the magnetic atoms *A* (*A* = Fe, Mn and Cr). It is clearly seen that the 3*d* electrons of Mn in the LMO/BTO superstructure increase and these electrons come from the BTO part. It is also found that the 3*d* electrons of Fe and Cr decrease and the lost electrons are transferred to the BTO region. The charge transfer between the BTO and LAO regions has little effect on the magnetic configurations of the superstructures. The reason for the change of magnetic orders could be due to electron transfer between the *e_g_* and *t_2g_* states. When the ferroelectric polarization changes from +*P* to −*P*, we found that the electrons transferred from the *t_2g_* orbital to the *e_g_* orbital (approximately 0.14) lead to magnetic transition from A-AFM to ferromagnetic in the LMO/BTO system. In the LCO/BTO system, in fact, the *e_g_* electron shows a large reduction while the *t_2g_* electron shows a major increase compared with the bulk LCO case. The electrons transferred from the *e_g_* orbital to the *t_2g_* orbital (approximately 0.15) may be the reason for the C-AFM→G-AFM phase transition. The electron transfer in the LFO/BTO system is clearly much smaller with respect to the other two cases, indicating the unchanged G-AFM state in both the ±*P* cases. Polarization-induced distortion variation of oxygen octahedra may lead to further degeneracy of 3*d* orbitals and electron transfer between *e_g_* and *t_2g_*_._ It is worth noting that Aruta *et al.* (2009 ▸) have investigated the magnetic properties of the LaMnO_3_/SrMnO_3_ superstructure through an X-ray linear dichroism technique. They demonstrated that the AFM→FM transition could be attributed to the electron transfer of the partially occupied Mn-*e_g_* orbitals. Nevertheless, the reason for the control of magnetism in our superstructures still needs to be verified by future experiments.

It was found that robust manipulation of the magnetism, including the exchange interaction energy and magnetic ordering in LAO/BTO (*A* = Fe, Mn and Cr) superstructures, can be achieved by ferroelectric polarization. Along with the manipulation of the magnetism, the electronic structure was also significantly modified by polarization, and half metallicity was observed in LMO and LCO/BTO with the appearance of a 2D electron gas at the interface. The ferroelectric polarization of BTO changes the Fe—O, Mn—O and Cr—O bond lengths of layers at the interface. Since the original bond lengths and bond strengths are different among the LFO, LMO and LCO bulks, the bond length variations of the three superstructure systems are also different. These bond length changes will lead to the tilting of oxygen octahedra at the interface, causing structural and electronic reconstruction. Ferroelectric polarization can modulate the carrier concentration by introducing an accumulation of spin-polarized electrons and a depletion of holes near the interfaces, and thus can control the interface magnetic moments and net magnetization correspondingly. These superstructures are stable, controllable, easily grown and low-cost, promising future applications in spintronics, chemical gas sensing and information storage.

## 3. Conclusions   

Although some multiferroic materials have been extensively investigated, finding strong magnetoelectric couplings for the full control of magnetization remains challenging. Here, we have built LaFeO_3_/BaTiO_3_, LaMnO_3_/BaTiO_3_ and LaCrO_3_/BaTiO_3_ (001) superstructures as proof of the potential for robust control of the magnetism when these magnetic layers are coupled to ferroelectric polarizations. Both superexchange and double-exchange interactions exist in these superstructures. The superexchange interaction can be found in the LMO/BTO superstructure in the +*P* case, while the double-exchange interaction plays a role in the remaining five cases. The LFO/BTO system shows a G-type antiferromagnetic order for both polarization directions. The BTO region is insulating, and the conductivity of this superstructure is entirely controlled by the LFO films. For the +*P* condition, the LMO/BTO system exhibits a metallic character and has A-AFM order. The stronger superexchange interaction contributed by the *t*_2*g*_ states stabilizes the antiferromagnetic phase of the LMO/BTO superstructure. In contrast, under the *−P* condition, it changes to ferromagnetic due to the spin splitting of the mixed Mn-3*d* and O-2*p* states. The LMO/BTO superstructure finally acquires half-metallic and semi-metallic character, which may be a result of the strong spin polarization. A maximal change of 99.7% of the net magnetization can be achieved by switching the ferroelectric polarization. Upon polarization switching, the magnetic moment of Mn in particular shows significant modulations, as listed in Table 1 ▸. The TiO_2_/LaO/MnO_2_ interface acts as a magnetic switch, which leads to a large variation in the magnetic moment and thus, the largest magnetoelectric effect among the superstructures. The LCO/BTO system can retain the G- and C-AFM configurations in the *−P* and *+P* cases, respectively. The magnetism variations of the three superstructures are mainly due to valence-state changes of the Fe/Mn/Cr ions and charge transfer among the Ti, O and Fe/Mn/Cr ions. The strong magnetoelectric coupling mediated by the interfacial effect enables full control of the magnetism. The LFO/BTO and LCO/BTO superstructures show a metal-insulator transition when the polarization of BTO reverses. The direction change of the ferroelectric polarization leads to electron transfer between the *e_g_* and *t_2g_* orbitals, which determines the variation of magnetic order of the three superstructures. The robust control of magnetism demonstrated in this article will provide a feasible scheme for experimental work.

## 4. Related literature   

The following references are cited in the supporting information: An *et al.* (2017 ▸); Betancourt *et al.* (2017 ▸); Blöchl (1994 ▸); Dabaghmanesh *et al.* (2017 ▸); Ding *et al.* (2010 ▸); Dudarev *et al.* (1998 ▸); Elemans *et al.* (1971 ▸); Fang & Nagaosa (2004 ▸); Hashimoto *et al.* (2010 ▸); He *et al.* (2010 ▸); Koehler & Wollan (1957 ▸); Kotomin *et al.* (2005 ▸); Li *et al.* (2017 ▸); Monkhorst & Pack (1976 ▸); Muñoz *et al.* (2004 ▸); Perdew *et al.* (1996 ▸); Pinsard-Gaudart *et al.* (2001 ▸); Prado-Gonjal *et al.* (2011 ▸); Scafetta *et al.* (2014 ▸); Selbach *et al.* (2012 ▸); Shein (2005 ▸); Siemons *et al.* (2007 ▸); Sushko *et al.* (2013 ▸); Yang *et al.* (1999 ▸).

## Supplementary Material

Supporting Information. DOI: 10.1107/S205225251801624X/lt5015sup1.pdf

## Figures and Tables

**Table 1:** The calculated energy difference (per Fe/Mn/Cr atom) between the reference ferromagnetic (FM) and antiferromagnetic (AFM) states *m*_1_ and *m*_2_ are the local magnetic moments for *A* cations using Wigner–Seitz spheres. *M* is the net magnetization. All moments are in units of μ_B_. G-AFM = G-type antiferromagnetic, C-AFM = C-type antiferromagnetic and A-AFM = A-type antiferromagnetic.

Superstructure	Ferroelectric	Order	*ΔE* (meV)	*m*_1_	*m*_2_	*M*
LaFeO_3_/BaTiO_3_	+*P*	FM	0	4.26	4.16	4.75
	+*P*	G-AFM	−152.26	4.13	−4.07	−0.01
	−*P*	FM	0	4.31	4.29	4.88
	−*P*	G-AFM	−247.87	4.16	−4.14	0
LaMnO_3_/BaTiO_3_	+*P*	FM	0	3.59	3.27	3.43
	+*P*	A-AFM	−30.48	3.24	−3.55	−0.01
	−*P*	FM	−42.45	3.58	3.77	3.82
	−*P*	A-AFM	0	3.77	−3.37	0.20
LaCrO_3_/BaTiO_3_	+*P*	FM	0	2.77	2.52	2.82
	+*P*	C-AFM	−45.28	2.76	−2.47	0.03
	−*P*	FM	0	2.80	2.78	2.86
	−*P*	G-AFM	−95.95	2.79	−2.78	0

**Table 2:** The number of electrons for the 3*d* orbitals (both *e_g_* and *t_2g_*) of the magnetic atoms in the LAO (*A* = Fe, Mn and Cr) bulks and the LAO/BTO superstructures

Materials	Ferroelectric	Order	*e_g_*	*t_2g_*	3*d* orbital
LaFeO_3_	0	G-AFM	2.17	3.82	5.99
LaFeO_3_/BaTiO_3_	+*P*	G-AFM	2.52	3.20	5.72
	−*P*	G-AFM	2.57	3.16	5.73
LaMnO_3_	0	A-AFM	1.87	2.89	4.76
LaMnO_3_/BaTiO_3_	+*P*	A-AFM	1.75	3.16	4.91
	−*P*	FM	1.91	3.03	4.94
LaCrO_3_	0	G-AFM	1.53	2.60	4.13
LaCrO_3_/BaTiO_3_	+*P*	C-AFM	1.17	2.80	3.97
	−*P*	G-AFM	1.03	3.04	4.07
