# Supplementary material for: Robust manipulation of magnetism in LaAO3/BaTiO3 (A = Fe, Mn and Cr) superstructures by ferroelectric polarization
Source: IUCrJ. 2019 Jan 15;6(Pt 2):189–96. doi: 10.1107/S205225251801624X (PMC6400182; doi:10.1107/S205225251801624X)
Supplement: Supplementary file 1 [file m-06-00189-sup1.pdf]

IUCrJ

Volume 6 (2019)

Supporting information for article:

Robust manipulation of magnetism in La*A*O3/BaTiO3 (*A* = Fe, Mn and Cr) superstructures by ferroelectric polarization

Dong Chen, Guangbiao Zhang, Zhenxiang Cheng, Shuai Dong and Yuanxu Wang

Supporting Information

Robust manipulation of magnetism in $LaAO_3/BaTiO_3$ (A = Fe, Mn, and Cr) superstructures by ferroelectric polarization

Dong Chen [a], Guangbiao Zhang [a], Zhenxiang Cheng [a,b]*, Shuai Dong [c], and Yuanxu Wang [a]*

[a] Institute for Computational Materials Science, School of Physics and Electronics, Henan University, Kaifeng, 475004, P. R. China,

[b] Institute for Superconducting and Electronic Materials, Australian Institute of Innovative Materials, University of Wollongong, NSW 2500, Australia,

[c] School of Physics, Southeast University, Nanjing, 211189, P. R. China

I. Calculation method

The generalized gradient approximation plus on-site Coulomb correction (GGA+*U*) scheme was used to describe the exchange-correlation potential with the famous Perdew-Burke-Ernzerhof (PBE) functional [1], as implemented in the Vienna ab initio simulation package (VASP5.3.5) [2]. The database of the pseudo-potentials is created in 06 September 2000. The Hubbard interaction $U_{eff} = U - J$ where U is the on-site Coulomb interaction parameter and J is the on-site exchange interaction parameter, is represented using the Dudarev approach [3] to properly capture the physical picture of a strongly correlated system. For simplicity, we use U instead of U_{eff} in the following paper, while the procedure for determining U is detailed in Section 3. With plane waves up to the energy-cutoff of 500 eV, the projector-augmented wave (PAW) method [4] is adopted to treat the valence states of the elements Ba, Ti, La, Fe, Cr, Mn, and regular O as $5s^2 5p^6 6s^2$, $3p^6 3d^2 4s^2$, $5s^2 5p^6 5d^1 6s^2$, $2p^6 3d^6 4s^2$, $3p^6 3d^5 4s^1$, $3p^6 3d^5 4s^2$, and $2s^2 2p^4$, respectively. For the superstructure systems, the bottom two units of BTO are fixed during the optimization to mimic a thick substrate, hence to maintain a relatively large FE-*P* of BTO. The internal atomic coordinates for the other layers are fully relaxed to reach the ground state. To obtain well converged results, we use 9×9×7 and 5×5×1 (11×11×1) Γ-centered Monkhorst-Pack *k*-points [5] for the 20 atom bulk compounds and 60 atoms SLs (density of states (DOS) calculations), respectively. The convergence tolerances are: difference in total energy within 10^{-6} eV/atom and maximum Hellmann-Feynman force less than 0.01 eV/Å. The spin-orbit coupling, which is weak and not important in the above-mentioned superstructures, is ignored in our DFT calculations.

II. Bulk properties of $LaAO_3$ (A = Fe, Mn, AND Cr)

TABLE S1 Structural parameters of $LaAO_3$ (A = Fe, Mn, and Cr) compared with the experimental data and previous theoretical works

Materials	ΔE (meV)	Method	Lattice constants a, b, c (Å)			m (μ_B/atom)	E_g (eV)
$LaFeO_3$	-286.5(G-AFM)	This work	5.5715	5.6248	7.9039	4.14	2.69
	-104.6(A-AFM)	Expt.[6]	5.5600	5.5600	7.8500	4.06 [7]	2.52 [7]
	-203.6(C-AFM)	Expt. [8]	5.5570	5.5650	7.8540	4.1 [9]	2.1 [9]
	0 (FM)	Expt. [10]	5.5500	5.5700	7.8500	Expt. [11] 4.6±0.2	Expt.[12] 2.34
$LaMnO_3$	-60.4(A-AFM)	This work	5.5713	5.8498	7.6955	3.64	0.98
	46.8(C-AFM)	Expt. [13]	5.5320	5.7420	7.6680	3.87 [14]	1.18 [15]
	265.6(G-AFM)	Expt. [16]	5.5397	5.4891	7.7928	3.8 [9]	1.0 [9]
	0 (FM)	Expt. [17]	5.5398	5.7488	7.6946	Expt. [18] 3.7±0.1	Expt. [14] 1.7
$LaCrO_3$	-85.6(G-AFM)	This work	5.5505	5.5761	7.8551	2.79	2.81
	-31.1(A-AFM)	Expt. [16]	5.5163	5.4789	7.7616	2.9 [9]	2.6 [9]
	-61.9(C-AFM)	Calc. [19]	5.5600	5.5600	7.8400	2.82 [19]	2.65 [19]
	0 (FM)	Expt. [20]	5.5148	5.4875	7.7515	Expt. [11] 2.8±0.2	Expt. [21] 2.8

We first tested and analysed the optimized energy difference ΔE, lattice parameters a, b, c, magnetic moment m, and band gap E_g of the orthorhombic $LaAO_3$ (A = Fe, Mn, and Cr) bulk materials, as illustrated in Table S1. According to our tests, the U was set to be 5 eV (3 eV) for the 3d orbital of Fe (Cr). According to the results of An et al. [22], if the famous Dudarev implementation [3] is adopted for the LMO bulks, the ground state is always FM after geometry optimization, regardless of the value of U_{eff} and the choice of functional such as PBE or PBE for solids (PBEsol) [22]. In order to obtain accurate results for LMO bulks, we used the Liechtenstein approach combined with the appropriate U and J (U = 2.7 eV and J =1.0 eV) [23]. In agreement with the experiments [9, 11], the lowest energies of $LaFeO_3$, $LaMnO_3$, and $LaCrO_3$ correspond to the G-, A-, and G-type antiferromagnetic configurations, respectively. In Table S1, the calculated ground-state properties agree well with the experimental data and previous theoretical results [6-21], which demonstrates that such U values are suitable for LAO_3 (A = Fe, Mn, and Cr). The equilibrium lattice constants are generally overestimated by 0.2−1.0% compared with the experimental data, which is within the typical accuracy of GGA-PBE plus U calculations. The band gaps indicate that they are all semiconductors, while $LaCrO_3$ has the largest band gap compared with the other two compounds. The maximum errors between the calculated and

measured [11, 12, 14, 18, 21] lattice constants and the magnetic moments of $LaAO_3$ do not exceed 3.6% and 11.1%, respectively.

The calculated lattice constants a, b, and c, and the coordinates of the La, A, O atoms in the LAO (A = Fe, Mn, and Cr) part of our superstructures are given in Tables S2-S4.

Table S2 The coordinates of the La, Fe, O atoms in the LFO part and the lattice constants of the LFO/BTO superstructure

FE-*P*	atom	x	y	z	FE-*P*	atom	x	y	z
+*P*	La1	0.0088	0.0054	0.8449	-*P*	La1	0.0091	0.0097	0.8483
+*P*	La2	0.0063	0.9830	0.6893	-*P*	La2	0.9899	0.9833	0.6916
+*P*	La3	0.4936	0.4834	0.6891	-*P*	La3	0.4931	0.4856	0.6926
+*P*	La4	0.4920	0.5007	0.8448	-*P*	La4	0.4942	0.4938	0.8476
+*P*	Fe1	0.5075	0.0024	0.7607	-*P*	Fe1	0.5052	0.9905	0.7696
+*P*	Fe2	0.9949	0.5021	0.7606	-*P*	Fe2	0.9926	0.5021	0.7696
+*P*	Fe3	0.4998	0.0005	0.9164	-*P*	Fe3	0.4969	0.0010	0.9197
+*P*	Fe4	0.0007	0.5011	0.9164	-*P*	Fe4	0.0021	0.4971	0.9197
+*P*	O1	0.0402	0.4887	0.8347	-*P*	O1	0.0467	0.4549	0.8445
+*P*	O2	0.4615	0.0021	0.8348	-*P*	O2	0.9652	0.5260	0.6927
+*P*	O3	0.7039	0.3015	0.7620	-*P*	O3	0.9652	0.5260	0.6927
+*P*	O4	0.2837	0.7223	0.7494	-*P*	O4	0.4514	0.0397	0.8445
+*P*	O5	0.2195	0.2229	0.7484	-*P*	O5	0.7057	0.2937	0.7699
+*P*	O6	0.7991	0.8018	0.7361	-*P*	O6	0.2964	0.7030	0.7676
+*P*	O7	0.2445	0.7557	0.9101	-*P*	O7	0.2098	0.2073	0.7572
+*P*	O8	0.7527	0.2477	0.9051	-*P*	O8	0.7944	0.7917	0.7788
+*P*	O9	0.7481	0.7474	0.9042	-*P*	O9	0.2398	0.7581	0.9182
+*P*	O10	0.2563	0.2561	0.9113	-*P*	O10	0.7575	0.2407	0.9169
+*P*	O11	0.9920	0.5018	0.9910	-*P*	O11	0.7418	0.7418	0.9111
+*P*	O12	0.5083	0.9985	0.9909	-*P*	O12	0.2585	0.2583	0.9269
+*P*	*a*=0. 5599 nm		*b*=0.5595 nm	*c*=2.4517 nm	-*P*	*a*=0.5588 nm		*b*=0.5588 nm	*c*=2.4549 nm

Table S3 The coordinates of the La, Mn, O atoms in the LMO part and the lattice constants of the LMO/BTO superstructure

FE-*P*	atom	x	y	z	FE-*P*	atom	x	y	z
+*P*	La1	0.9999	0.0014	0.8442	-*P*	La1	0.0071	0.0147	0.8478
+*P*	La2	0.0012	0.9931	0.6870	-*P*	La2	0.9947	0.9823	0.6908
+*P*	La3	0.4969	0.4926	0.6870	-*P*	La3	0.5002	0.4824	0.6915
+*P*	La4	0.5004	0.5014	0.8441	-*P*	La4	0.4960	0.5032	0.8475
+*P*	Mn1	0.5012	0.0011	0.7626	-*P*	Mn1	0.5054	0.9971	0.7693
+*P*	Mn2	-0.0010	0.5013	0.7626	-*P*	Mn2	0.9968	0.5034	0.7692
+*P*	Mn3	0.4977	0.0007	0.9135	-*P*	Mn3	0.4954	0.0018	0.9204
+*P*	Mn4	0.0023	0.5002	0.9135	-*P*	Mn4	0.0046	0.5000	0.9206

+*P*	O1	0.0073	0.4996	0.8363	-*P*	O1	0.0468	0.4748	0.8448
+*P*	O2	0.4927	0.0015	0.8363	-*P*	O2	0.9581	0.5184	0.6922
+*P*	O3	0.7002	0.3016	0.7596	-*P*	O3	0.5432	0.9909	0.6923
+*P*	O4	0.2962	0.7055	0.7558	-*P*	O4	0.4553	0.0188	0.8449
+*P*	O5	0.2046	0.2061	0.7552	-*P*	O5	0.7080	0.2932	0.7730
+*P*	O6	0.8006	0.8021	0.7602	-*P*	O6	0.2951	0.7060	0.7638
+*P*	O7	0.2438	0.7569	0.9075	-*P*	O7	0.2084	0.2075	0.7588
+*P*	O8	0.7553	0.2455	0.9058	-*P*	O8	0.7929	0.7911	0.7775
+*P*	O9	0.7450	0.7457	0.9056	-*P*	O9	0.2448	0.7550	0.9206
+*P*	O10	0.2563	0.2569	0.9078	-*P*	O10	0.7530	0.2476	0.9161
+*P*	O11	0.9975	0.5006	0.9888	-*P*	O11	0.7467	0.7475	0.9141
+*P*	O12	0.5025	0.0001	0.9888	-*P*	O12	0.2543	0.2545	0.9246
+*P*	*a*=0. 5625 nm		*b*=0.5625 nm	*c*=2.4276 nm	-*P*	*a*=0.5590 nm		*b*=0.5588 nm	*c*=2.4581 nm

Table S4 The coordinates of the La, Cr, O atoms in the LCO part and the lattice constants of the LCO/BTO superstructure

FE-*P*	atom	x	y	z	FE-*P*	atom	x	y	z
+*P*	La1	0.9926	0.0039	0.8408	-*P*	La1	0.9961	0.0094	0.8472
+*P*	La2	0.0018	0.9843	0.6811	-*P*	La2	0.9919	0.9801	0.6894
+*P*	La3	0.4964	0.4844	0.6812	-*P*	La3	0.5056	0.4845	0.6894
+*P*	La4	0.5078	0.5053	0.8406	-*P*	La4	0.5068	0.5114	0.8466
+*P*	Cr1	0.5039	0.0017	0.7587	-*P*	Cr1	0.4939	0.9981	0.7694
+*P*	Cr2	0.9963	0.5017	0.7587	-*P*	Cr2	0.0049	0.4955	0.7694
+*P*	Cr3	0.4836	0.0032	0.9122	-*P*	Cr3	0.4911	0.0029	0.9190
+*P*	Cr4	0.0164	0.5003	0.9122	-*P*	Cr4	0.0089	0.4999	0.9190
+*P*	O1	0.0305	0.4976	0.8335	-*P*	O1	0.0308	0.4913	0.8443
+*P*	O2	0.4693	0.0035	0.8335	-*P*	O2	0.9570	0.5133	0.6929
+*P*	O3	0.7174	0.2836	0.7609	-*P*	O3	0.5423	0.9949	0.6929
+*P*	O4	0.2810	0.7203	0.7485	-*P*	O4	0.4682	0.0034	0.8443
+*P*	O5	0.2196	0.2209	0.7472	-*P*	O5	0.7065	0.2916	0.7739
+*P*	O6	0.7829	0.7838	0.7619	-*P*	O6	0.2927	0.7057	0.7628
+*P*	O7	0.2473	0.7538	0.9097	-*P*	O7	0.2068	0.2062	0.7606
+*P*	O8	0.7512	0.2502	0.9017	-*P*	O8	0.7917	0.7907	0.7768
+*P*	O9	0.7487	0.7502	0.9011	-*P*	O9	0.2454	0.7547	0.9206
+*P*	O10	0.2526	0.2538	0.9106	-*P*	O10	0.7528	0.2479	0.9156
+*P*	O11	0.9901	0.5014	0.9876	-*P*	O11	0.7472	0.7482	0.9149
+*P*	O12	0.5098	0.9999	0.9876	-*P*	O12	0.2544	0.2548	0.9222
+*P*	*a*=0.5622 nm		*b*=0.5622 nm	*c*=2.4311 nm	-*P*	*a*=0.5606 nm		*b*=0.5604 nm	*c*=2.4573 nm

Table S5 The normal modes Q_2 and Q_3 of the Jahn-Teller distortions, the octahedral tilt angle θ and the octahedral rotation angle φ of the

LAO/BTO (A = Fe, Mn, and Cr) superstructures

SL	FE-*P*	Layer	Q_2 /Å	Q_3 /Å	ϑ /degree	φ /degree
$LaFeO_3$/ $BaTiO_3$	+*P*	upper layer	0.027	0.144	7.45	0.76
		lower layer	0.066	0.134	4.51	9.93
	-*P*	upper layer	0.027	0.282	11.38	1.95
		lower layer	0.036	0.071	6.25	9.71
$LaMnO_3$/ $BaTiO_3$	+*P*	upper layer	0.031	0.037	1.20	1.23
		lower layer	0.010	0.139	1.76	11.17
	-*P*	upper layer	0.054	0.266	9.10	0.85
		lower layer	0.047	0.042	7.07	9.38
$LaCrO_3$/ $BaTiO_3$	+*P*	upper layer	0.206	0.049	4.25	0.37
		lower layer	0.063	0.105	5.37	7.27
	-*P*	upper layer	0.101	0.324	4.31	0.73
		lower layer	0.057	0.042	7.87	9.46

In Table S5, the Jahn-Teller distortions around the Fe/Mn/Cr atoms at the upper layer (Layer 12) and lower layer (Layer 10) can be described by normal modes Q_2 and Q_3, which can be obtained by $Q_2 = (1/\sqrt{2})(X_1 - X_4 - Y_2 + Y_5)$ and $Q_3 = (1/\sqrt{6})(2Z_3 - 2Z_6 - X_1 + X_4 - Y_2 + Y_5)$ [15]. In these two equations, the *X*, *Y*, and *Z* are the coordinates of the surrounding oxygen atoms. The experimental values of Q_2 and Q_3 for the $LaMnO_3$ (calculated results of $LaFeO_3$/$LaCrO_3$) bulks are 0.074 (0.006/0.001) and 0.413 Å (0.316/0.004 Å), respectively [15, 24]. The Q_2 and Q_3 of LMO/BTO are greatly reduced, while these two modes of LFO/BTO and LCO/BTO are significantly enhanced compared with the corresponding bulks. We found that the lattice distortion of LCO/BTO is the most pronounced and the Jahn-Teller distortions of LFO/BTO and LMO/BTO are relatively slight. We found that the effect of Jahn-Teller on the magnetism variation is very small and negligible.

In addition to the shape distortions, which modify the A-O (A = Fe, Mn, and Cr) bond lengths, rotations and tiltings of the AO_6 units buckle the octahedral framework along different axes and play a key role in determining the physical properties of perovskite SLs. As shown in Table S5, the octahedral tilt angle along the *z*-axis is defined as $\theta = (180° - \Theta)/2$, where Θ is the bond angle of A-O-A (A = Fe, Mn, and Cr) along the *z*-axis. The rotation angle in the *x*-*y* plane can be defined as $\varphi = (90° - \Omega)/2$, where Ω is the angle among the three oxygen ions between two corner-shared oxygen octahedra in the *x*-*y* plane [25]. θ and φ represent the tilt angle and the rotation angle of the oxygen octahedra, respectively [26]. When the polarization reverses from +*P* to –*P*, the tilts are almost suppressed by the FE-*P* and the out-of-plane rotation φ becomes the

dominant octahedral distortion in the LMO/BTO superstructure. At the same time, the magnetic moments of Mn atoms decrease very slightly (see Table 1). It is generally believed that the smaller the θ and φ is, the closer the A-O-A bond angle (A = Fe, Mn, and Cr) to 180°. This is favorable for the formation of FM state. Both the large variation of θ and φ in the LMO/BTO SL and the small change of θ and φ in LFO/BTO and LCO/BTO SLs do not play an important role in the magnetic moment variations. The change of magnetism in the three LAO/BTO (A = Fe, Mn, and Cr) SLs could be caused by the electron transfer between the e_g and t_{2g} orbitals of the magnetic ions.

III. Electronic structures

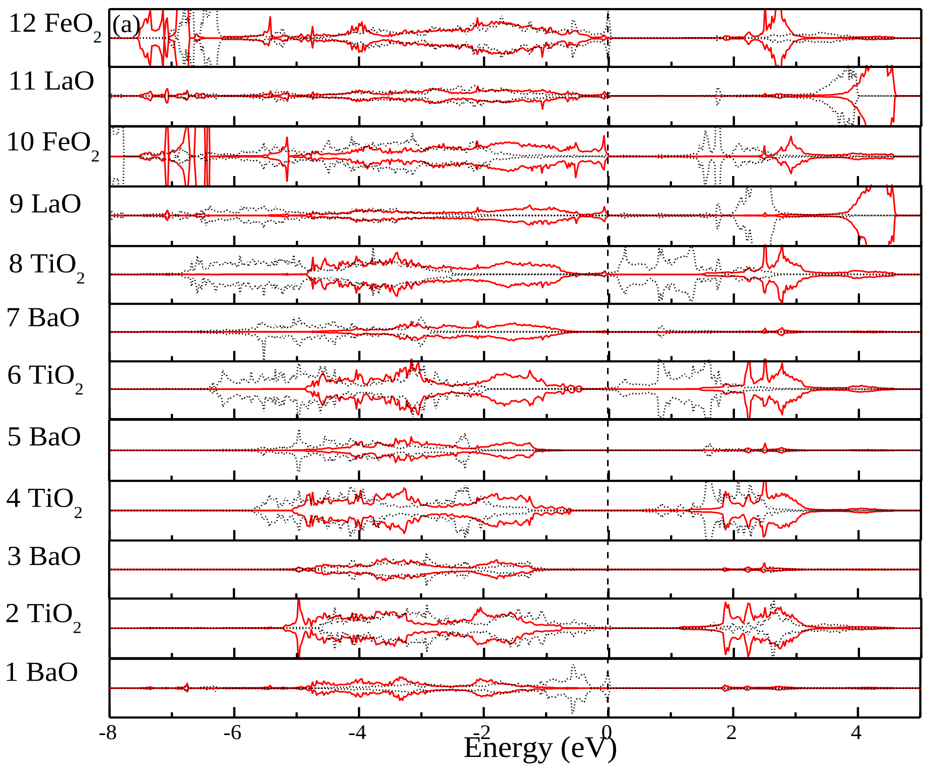

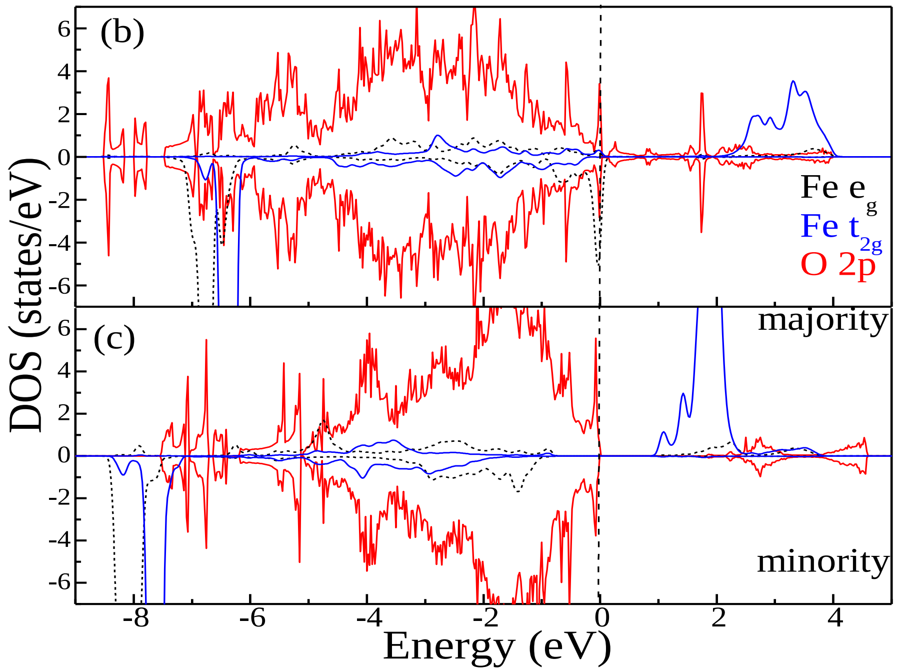

Figure S1. (a) The layer-resolved density of states (LDOS) of the LFO/BTO (001) superstructure (black line: $+P$, red line: $-P$). (b) The projected density of states (PDOS) of the LFO layers for the $+P$ case. (c) the PDOS of the LFO layers for the $-P$ case. (The Fermi level E_F is located at 0 eV and indicated by the vertical dashed line.)

The layer-resolved density of states (LDOS) of LFO/BTO and the projected density of states (PDOS) of LFO are plotted in Fig. S1(a) and Fig. S1(b) and S1(c), respectively. The black-dotted line (red solid line) represents the $+P$ ($–P$) case. The interface of BTO and LFO is between the eighth and ninth layers. The DOS near the Fermi level determines the electronic properties, so we just pay attention to the DOS between -8 and 5 eV. The spin-up and spin-down LDOSs are almost symmetric, which indicates that the system can maintain its AFM configuration in both $\pm P$ cases. Obvious gaps can be found at the Fermi level, indicating a semiconductive character for LFO/BTO. In the BTO region (Layers 1 to 8), the top of the valence band (VB) shows a downward shift to lower energy when the BTO layers are close to the interface (under the $+P$ condition). In the LFO region (Layers 9 to 12), the top of the VB under the $+P$ condition shifts downward when the LFO layers approach the interface, while it does not show any visible shift in both the LFO and the BTO layers for the $–P$ case. This suggests that the FE polarization ($–P$) is weak in the LFO/BTO (001) SL, which does not generate a strong internal electric field.

The Fe/Mn/Cr atoms are thus in an octahedral environment, which induces an energetic splitting of the 3d orbitals into a t_{2g} threefold-degenerate low-energy set and an e_g twofold-degenerate high-energy one (ideal case). Fig. S1(b) and S1(c) presents the partial DOS of the Fe-t_{2g} (solid) and Fe-e_g (dotted) symmetries, in combination with the O-2p states. Among the La, Fe, and O atoms, noticeable changes are found only in these orbitals. Both e_g and t_{2g} electrons are present throughout the VB region, and hence, they participate in the covalent bonding with

oxygen. The structures well below the Fermi level are composed of the majority and minority spin states of O-*p* hybridized with the Fe-e_g and Fe-t_{2g} states. As shown in Fig. S1(b), the DOS at the Fermi energy, E_F, consists of mainly the O-2*p* state and the spin-down e_g state, as well as a small contribution of spin-up Fe-t_{2g} orbitals. Strongly hybridized states between the Fe and O atoms are found in both spin-up and spin-down channels. In Fig. S1(c), the spin-down e_g band is fully occupied, while the spin-up e_g and t_{2g} states are located well above E_F. For the –*P* case, the LFO/BTO system is totally insulating. The Fe-3*d* and O-2*p* states are shifted toward lower and higher energy regions, respectively. Correspondingly, the hybridization between Fe and O is weakened compared with the +*P* case.

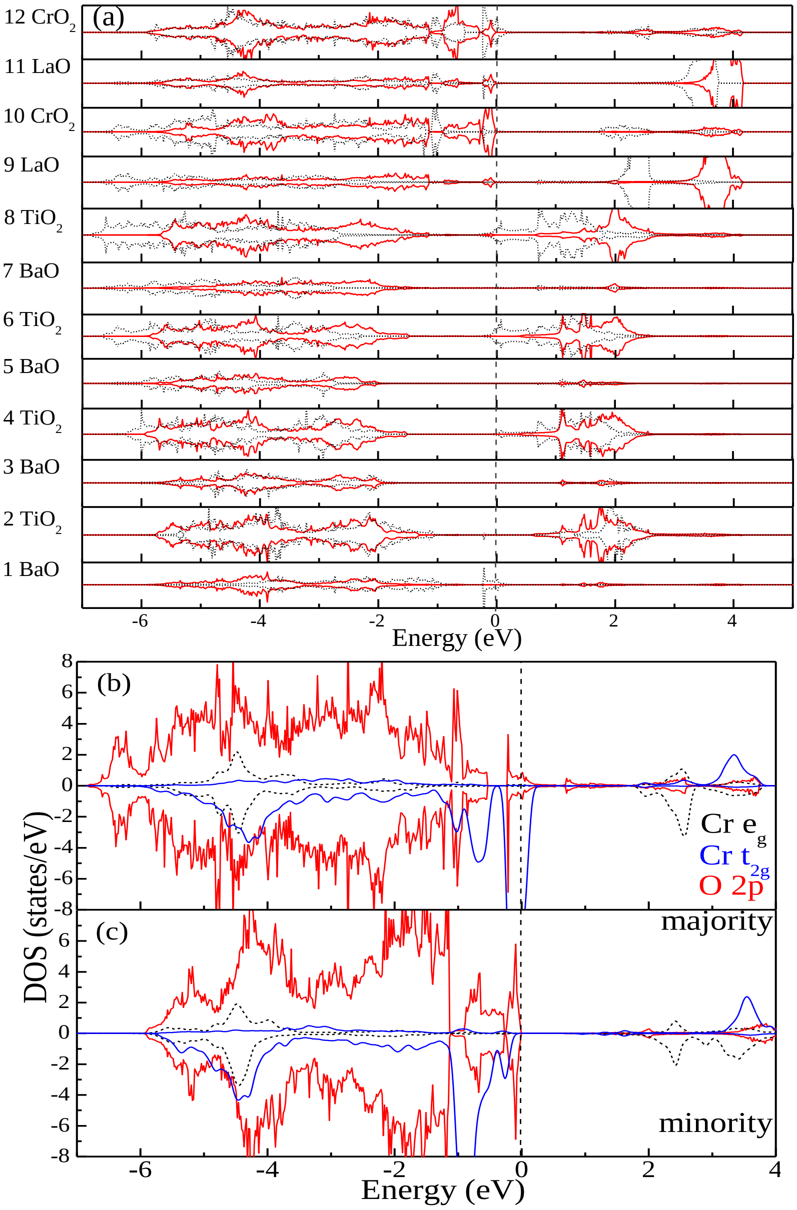

Figure S2. (a) Layer-resolved density of states (LDOS) of the LCO/BTO (001) superstructure (black line: $+P$, red line: $-P$). (b) The projected density of states (PDOS) of the LCO layers for the $+P$ case. (c) The PDOS of the LCO layers for the $-P$ case. (The Fermi level E_F is located at 0 eV and indicated by the vertical dashed line.)

According to Fig. S2(a), there is no spin splitting between the spin-up and spin-down channels, so the LDOS is symmetric. In the +*P* case, the BTO layers are semiconductive while the LCO region is metallic. Therefore, it is easier for the itinerant electrons to move along the *a* and *b* axes within the LCO layers rather than move across the BTO region along the *c* axis. A two-dimensional electron gas (2DEG) can be observed near the E_F of the BaO/CrO_2 interface. For the +*P* case, the top of the VB shows a downward shift from the bottom BaO layer to the interface with the TiO_2 layer (eighth layer). For the –*P* case, the top of the valence band shows a downward shift first (Layer 1 to Layer 5), and then the valence bands shift upward from Layers 6 to 8. These results suggest that the reversal of *P* changes the internal electrical field of the superstructure. Nevertheless, the top of the valence band shows no macroscopic shift in the LCO film in either case.

Our PDOS predicts that there is considerable *p* and *d* characters that appears at the top of the VB. It is clear that the spin-down DOS of Cr-3*d* is much stronger than the spin-up DOS. As shown in Fig. S2(b), the O-*p*, Cr-e_g, and Cr-t_{2g} states are mainly located in the energy range of -7−0 eV. A large peak near the E_F can be seen for the spin-down channel of the Cr-t_{2g} states, while the CB DOS is located far away from the Fermi level. The CB DOS is predominantly occupied by the O-2*p* state, and the majority Cr-t_{2g} and minority Cr-e_g states. In Fig. S2(c), it is interesting to see that the width of the PDOS is much narrower than that in Fig. S2(b), indicating that the chemical bonding of the LCO/BTO system in the –*P* case is weakened compared with the +*P* case. The PDOS shows an apparent upward shift from the +*P* to the –*P* case, which implies that the electrons at E_F are transferred. It is clearly seen in Fig. S2(b) and S2(c) that the Cr-e_g and Cr-t_{2g} bands are strongly hybridized with O-2*p* states around the Fermi level. This strong interaction leads to the formation of bonding states.

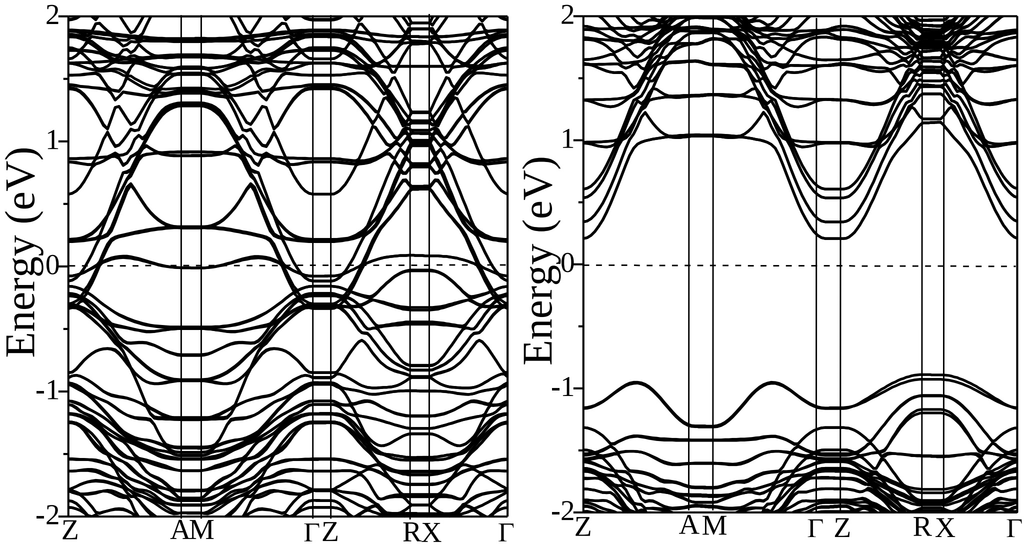

(a) (b)

Figure S3. Band structures of LFO/BTO superstructures in (a) the +*P* case and (b) the –*P* case. The dashed line represents the Fermi level.

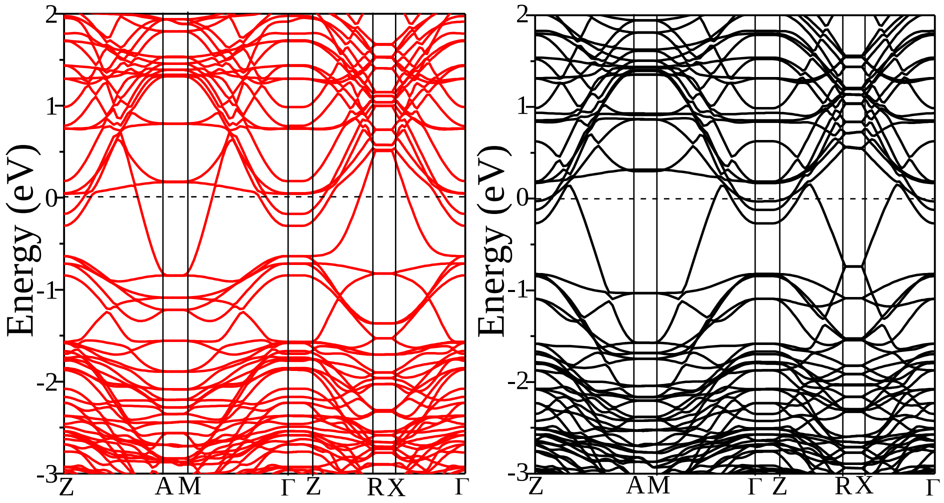

(a) (b)

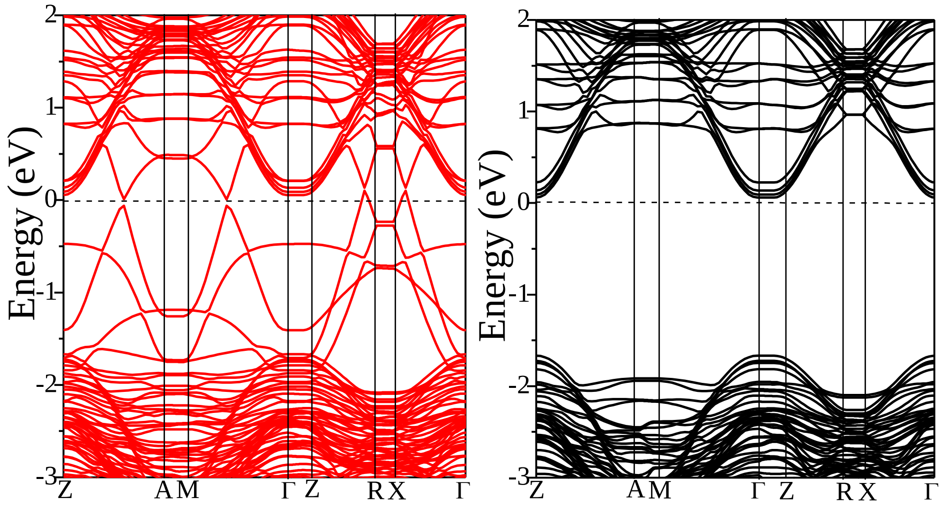

(c) (d)

Figure S4. Band structures of LMO/BTO superstructures with (a) spin-up for the +*P* case; (b) spin-down for the +*P* case; (c) spin-up for the –*P* case; (d) spin-down for the –*P* case.

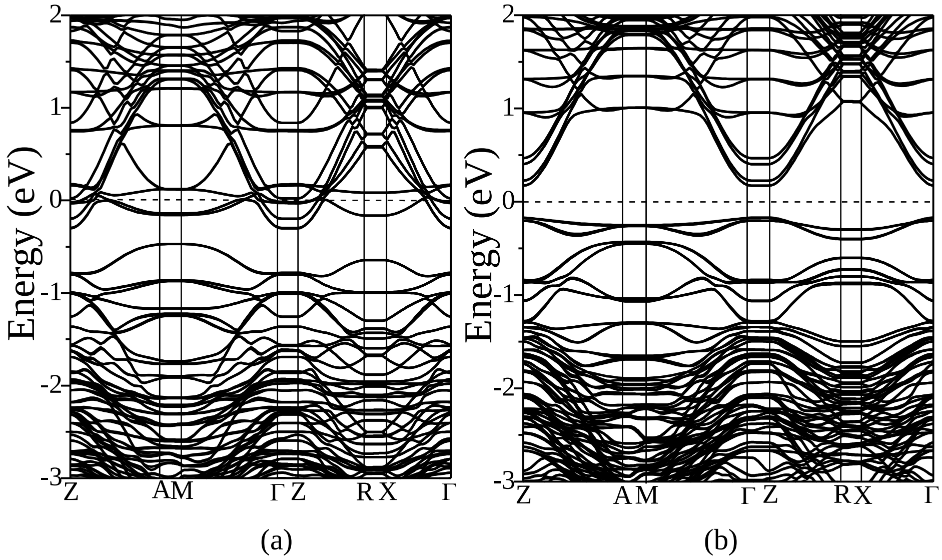

Figure S5. The band structures of LCO/BTO superstructures for (a) the +*P* case and (b) the –*P* case.

As illustrated in Fig. S3(a) and S3(b), the LFO/BTO system is metallic and semiconductive with an indirect band gap of 1.12 eV in the +*P* and –*P* cases, respectively. We can see that the LFO/BTO system has a metal-insulator transition, which agrees well with the DOS results. Because of the FM order of LMO/BTO, the band structures of the spin-up and spin-down channels are given in Fig. S4. In Fig. S4(a) and S4(b), both two channels show metallic characteristics. Fig. S4(c) shows two Dirac points located along the Z–A and M–Γ directions, indicating topological features of the bands. In Fig. S4(d), a direct band gap of 1.61 eV at the Γ point can be seen. The bands exhibited in Fig. S5(a) cross the Fermi level, exhibiting a metallic character, whereas the band given in Fig. S5(b) is semiconducting with a direct band gap of 0.35 eV, showing a significant effect as the ferroelectric *P* reverses. The electron charge carriers have large effective masses; therefore their mobilities are very low along all directions.

IV. Exchange coupling studies

The exchange interactions can be obtained by mapping the total energies of each magnetic configuration, *E(FM)*, *E(A)*, *E(C)* and *E(G)*, to the classical Heisenberg model. The intraplane J_{ab} and interplane J_c coupling constants of LAO/BTO (A = Fe, Mn, and Cr) superstructures can be described as [27]

$$J_{ab} = (1/8S^2)[E(FM) - E(G) - E(A) + E(C)], \quad (1)$$

$$J_c = (1/4S^2)[E(FM) - E(G) - E(C) + E(A)], \quad (2)$$

where S = 4.2, 3.6, and 2.8 μ_B are the calculated magnetic moments for the Fe, Mn, and Cr cations, respectively. The Curie temperature T_c (Néel temperature T_N) can be described as [28]

$$T_N\left(T_c\right) = T_3 / \ln(\frac{3\pi T_3}{4K}), \quad T_3 = 4\pi J/3, \quad J = \frac{E_{AFM} - E_{FM}}{zN_{FM}}, \quad (3)$$

where E_{FM} and E_{AFM} are the free energies of the FM and most stable AFM configurations, respectively. N_{FM} represents the number of magnetic atoms. J is the exchange interaction of all neighboring atoms. K represents the anisotropy constant, assumed to be 60 K. The coordination number z is 6 for the three-dimensional magnetic order [29].

TABLE S6 Exchange coefficients J_{ab}, J_c, Néel temperature T_N, and Curie temperature T_c of different SLs.

FE	Property	LFO/BTO	LMO/BTO	LCO/BTO
$+P$	J_{ab}	6.14 meV	-10.70 meV	4.27 meV
$+P$	J_c	2.43 meV	5.15 meV	2.43 meV
$-P$	J_{ab}	9.98 meV	-12.79 meV	6.79 meV
$-P$	J_c	8.13 meV	-5.35 meV	10.89 meV
$+P$	T_N/T_c	T_N =123.7 K	T_N =108.7 K	T_N =71.6 K
$-P$	T_N/T_c	T_N =168.4 K	T_c =132.1 K	T_N =95.6 K

J_{ab} and J_c are well-known to be sensitive, for example, to the variation in the A-O-A (A = Fe, Mn, and Cr) angle through the superexchange interactions. For the +*P* case of the LFO/BTO SL, J_{ab} is larger than J_c, which shows a two-dimensional (2D) character. When considering the –*P* case, the very close J_{ab} and J_c values show that the exchange interaction is nearly isotropic, which is in contrast to the other two SLs. J_{ab} and J_c are positive, indicating the ferromagnetic coupling in both the *a*-*b* plane and along the *c* axis. For the LMO/BTO system, the variation of exchange coupling is somewhat complicated. For the +*P* case, J_{ab} is negative, while J_c is positive, indicating the antiferromagnetic coupling in the *a*-*b* plane and the ferromagnetic coupling along the *c* axis. For the –*P* case, both J_{ab} and J_c are negative. The J_{ab} values are twice as large as the J_c, which reflects that the much greater strength of the exchange couplings in the *a*-*b* plane than those along the *c* axis. For the LCO/BTO SL, the two exchange couplings are both ferromagnetic. In the –*P* case, $J_c > J_{ab}$ means that the exchange coupling along the *c* axis is more stable than that in the *a*-*b* plane and vice versa. Although it is well known that the Curie temperature will be a little underestimated if the classical approximation to the Heisenberg model is used, the Curie temperature (T_c) and Néel temperature (T_N) calculated by the above-mentioned equations are all below room temperature, and hence, it is necessary to improve the values of T_c and T_N in experiments.

References

[1] Perdew, J. P., Burke, K. & Ernzerhof, M. (1996). *Phys. Rev. Lett.* **77**, 3865-3868.

[2] Kresse, G. & Furthmüller, J. (1996). *Phys. Rev. B,* **54**, 11169-11186.

[3] Dudarev, S. L., Botton, G. A., Savrasov, S. Y., Humphreys, C. J. & Sutton, A. P. (1998). *Phys. Rev. B,* **57**, 1505-1509.

[4] Blöchl, P. E. (1994). *Phys. Rev. B*, **50**, 17953-17979.

[5] Monkhorst, H. J. & Pack, J. D. (1976). *Phys. Rev. B*, **13**, 5188-5192.

[6] Ding, J. L., Lü, X. M., Shu, H. M., Xie, J. M. & Zhang, H. (2010). *Mater. Sci. Eng. B*, **171**, 31-34.

[7] Shein, I. R., Shein, K. I., Kozhevnikov, V. L. & Ivanovskiĭ, A. L. (2005). *Phys. Solid State*, **47**, 2082-2088.

[8] Acharya, S., Mondal, J., Ghosh, S., Roy, S. K. & Chakrabarti, P. K. (2010). *Mater. Lett.* **64**, 415-418.

[9] Yang, Z. Q., Huang, Z., Ye, L. & Xie, X. D. (1999). *Phys. Rev. B*, **60**, 15674-15682.

[10] Selbach, S. M., Tolchard, J. R., Fossdal, A. & Grande, T. (2012). *J. Solid State Chem.* **196**, 249-254.

[11] Koehler, W. C. & Wollan, E. O. (1957). *J. Phys. Chem. Solids*, **2**, 100-106.

[12] Scafetta, M. D., Cordi, A. M., Rondinelli, J. M. & May, S. J. (2014) *J. Phys.: Condens. Matter*, **26**, 505502.

[13] Kotomin, E. A., Evarestov, R. A., Mastrikov, Y. A. & Maier, J. (2005). *Phys. Chem. Chem. Phys.* **7**, 2346-2350.

[14] Muñoz, D., Harrison, N. M. & Illas, F. (2004). *Phys. Rev. B*, **69**, 085115.

[15] Hashimoto, T., Ishibashi, S. & Terakura, K. (2010). *Phys. Rev. B*, **82**, 045124.

[16] Prado-Gonjal, J., Arévalo-López, Á. M. & Morán, E. (2011). *Mater. Sci. Bull.* **46**, 222-230.

[17] Pinsard-Gaudart, L., Rodríguez-Carvajal, J., Daoud-Aladine, A., Goncharenko, I., Medarde, M., Smith, R. I. & Revcolevschi, A. (2001). *Phys. Rev. B*, **64**, 064426.

[18] Elemans, J. B. A. A., Van Larr, B., Van der Veen, K. R. & Loopstra, B. O. (1971). *J. Solid State Chem.* **3**, 238-242.

[19] Dabaghmanesh, S., Sarmadian, N., Neyts, E. C. & Partoens, B. (2017). *Phys. Chem. Chem. Phys.* **19**, 22870-22876.

[20] Siemons, M., Leifert, A. & Simon, U. (2007). *Adv. Funct. Mater.* **17**, 2189-2197.

[21] Sushko, P. V., Qiao, L., Bowden, M., Varga, T., Exarhos, G. J., Urban III, F. K., Barton, D. & Chambers, S. A. (2013). *Phys. Rev. Lett.* **110**, 077401.

[22] An, M., Weng, Y. K., Zhang, H. M., Zhang, J. J., Zhang, Y. & Dong, S. (2017). *Phys. Rev. B*, **96**, 235112.

[23] Lee, J. H., Delaney, K. T., Bousquet, E., Spaldin, N. A. & Rabe, K. M. (2013). *Phys. Rev. B*, **88**, 174426.

[24] Li, Y. R., Hou, Z. T., Wang, T. X., Li, Y., Liu, H. Y., Dai, X. F. & Liu, G. D. (2017). *J. Phys.: Conf. Series*, **827**, 012015.

[25] He, J., Borisevich, A., Kalinin, S. V., Pennycook, S. J. & Pantelides, S. T. (2010). *Phys. Rev. Lett.* **105**, 227203.

[26] Betancourt, J., Paudel, T. R., Tsymbal, E. Y. & Velev, J. P. (2017). *Phys. Rev. B*, **96**, 045113.

[27] Fang, Z. & Nagaosa, N. (2004). *Phys. Rev. Lett.* **93**, 176404.

[28] Yin, Y. W., Burton, J. D., Kim, Y. M., Borisevich, A. Y., Pennycook, S. J., Yang, S. M., Noh, T. W., Gruverman, A., Li, X. G., Tsymbal, E. Y. & Li, Q. (2013). *Nature Mater.* **12**, 397-402.

[29] Wei, L. Y., Lian, C. & Meng, S. (2017). *Phys. Rev. B,* **95**, 184102.
